# First report of paired ventral endites in a hurdiid radiodont

**DOI:** 10.1186/s40851-019-0132-4

**Published:** 2019-06-11

**Authors:** Stephen Pates, Allison C. Daley, Nicholas J. Butterfield

**Affiliations:** 1 Department of Zoology, University of Oxford, South Parks Road, Oxford, OX1 3PS UK; 2 Institute of Earth Sciences, University of Lausanne, CH-105 Lausanne, Switzerland; 3 Department of Earth Sciences, University of Cambridge, Downing Street, Cambridge, CB2 3EQ UK

**Keywords:** Radiodonta, Hurdiidae, *Ursulinacaris grallae*, Frontal appendages, Paired endites, Cambrian, Mount Cap Formation, Carrara Formation

## Abstract

**Background:**

Radiodonta, large Palaeozoic nektonic predators, occupy a pivotal evolutionary position as stem-euarthropods and filled important ecological niches in early animal ecosystems. Analyses of the anatomy and phylogenetic affinity of these large nektonic animals have revealed the origins of the euarthropod compound eye and biramous limb, and interpretations of their diverse feeding styles have placed various radiodont taxa as primary consumers and apex predators. Critical to our understanding of both radiodont evolution and ecology are the paired frontal appendages; however, the vast differences in frontal appendage morphology between and within different radiodont families have made it difficult to identify the relative timings of character acquisitions for this body part.

**Results:**

Here we describe a new genus of hurdiid, *Ursulinacaris*, from the middle Cambrian (Miaolingian, Wuliuan) Mount Cap Formation (Northwest Territories, Canada) and Jangle Limestone (Nevada, USA). *Ursulinacaris* has the same organisation as other hurdiid frontal appendages, with elongate endites on the first five podomeres in the distal articulated region and auxiliary spines on the distal margin of endites only. Unlike all other hurdiid genera, which possess a single row of elongated and blade-like ventral endites, this taxon uniquely bears paired slender endites.

**Conclusion:**

The blade-like endite morphology is shown to be a hurdiid autapomorphy. Two other frontal appendage characters known only in hurdiids, namely auxiliary spines on the distal margin of endites only, and elongate endites on the first five podomeres in the distal articulated region only, predate this innovation.

## Background

Radiodonta, as large nektonic predators in Palaeozoic oceans, were an important member of marine ecosystems and played a pivotal role in structuring these early animal communities. These stem euarthropod predators with raptorial appendages [1, 2], are a diverse and disparate group with over 25 species and 10 genera known from Africa, Australia, China, Europe, Greenland, and North America [3–11]. Recovered from deposits ranging from the Cambrian Series 2, Stage 3 to at least the Early Ordovician, and possibly even the Early Devonian, in age, these animals had body lengths from under 10 cm to around two meters [10–17]. The radiodont body plan consists of paired arthropodised frontal appendages that attach adjacent to radial mouthparts, with two compound eyes attached to the dorsal surface of the head with stalks. The body itself is metameric, bearing lateral flaps and setal blades [2].

Radiodonts have provided crucial information for understanding euarthropod evolution, for example the origin of the biramous limb (homologous to the ventral and dorsal flaps in hurdiids) and compound eye [10, 18, 19]. The paired frontal appendages have been shown to be homologous to the labrum in deuteropods, based on their morphological (adjacent to the mouthparts) and neuroanatomical (protocerebral) positions, and the pattern of labrum development [12, 20, 21].

As frontal appendages were used in feeding, the wide variety of known morphologies indicates that radiodonts occupied a number of niches in Cambrian ecosystems, including roles as primary consumers, sediment sifters and raptorial predators [7, 16, 18, 22, 23]. Frontal appendages are also critical for understanding radiodont internal relationships, as they have the highest preservation potential of all radiodont body parts (numerous taxa are only known from frontal appendages) and are character-rich, making them useful for phylogenetic analyses and taxonomy.

To date, all phylogenetic analyses (using both parsimony and Bayesian methods) exploring radiodont interrelationships have resolved two major clades of two radiodont families each. One clade is formed of the families Amplectobeluidae and Anomalocarididae, with the other comprising Hurdiidae and Tamisiocarididae [10, 12, 16, 17, 23].

## Identification of the shaft and distal articulated region

Central to our understanding of radiodont frontal appendages are the two major regions of this body part: the ‘shaft’ [14, 24], which has also been called the ‘peduncle’ in some studies (e.g. [16, 25]), and the ‘distal articulated region’ (defined by [24]). The boundary between these two regions of the frontal appendage can be identified by the degree of articulation between podomeres, an angle on the dorsal surface of the appendage, and/or the morphology and position of endites.

**Fig. 1:**
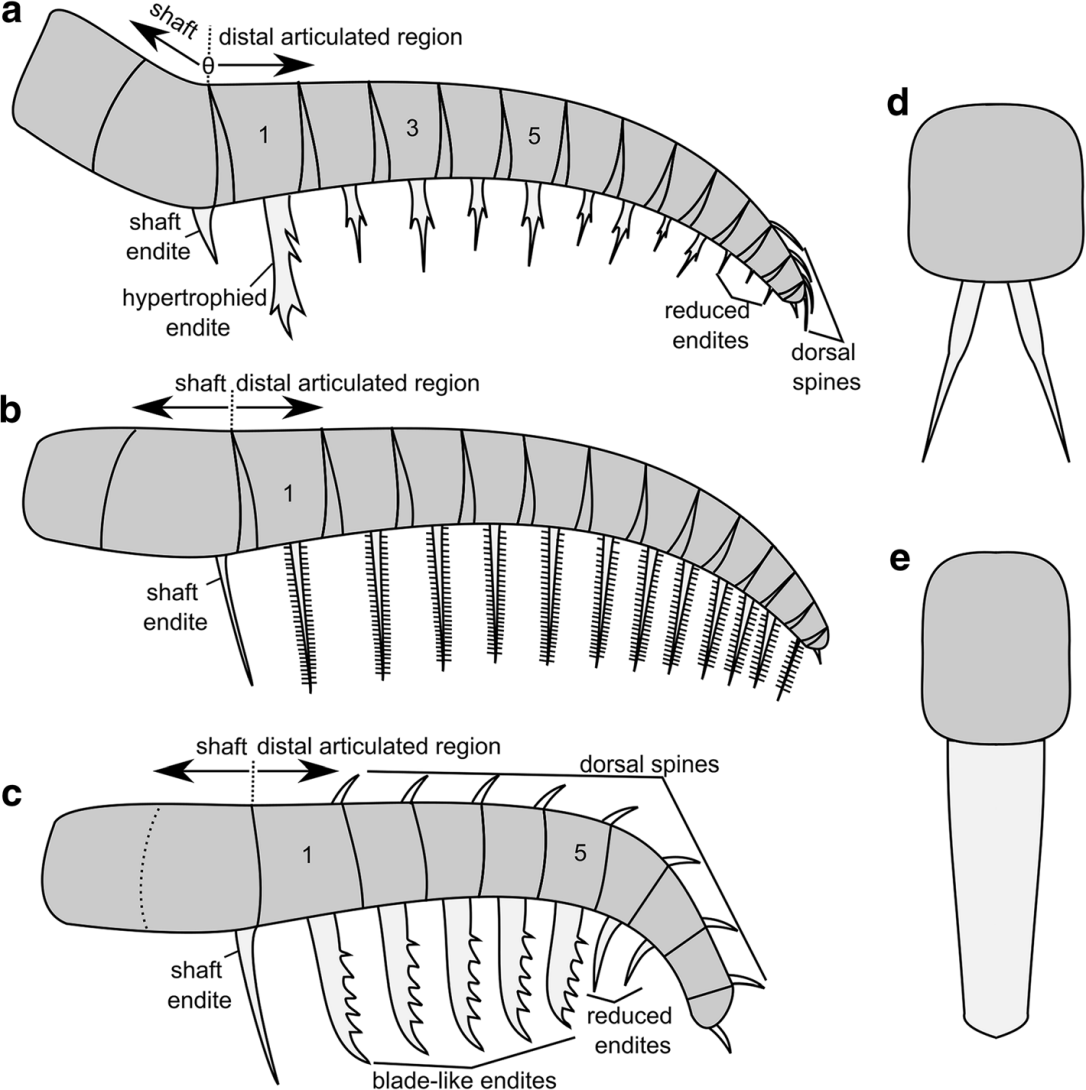
Schematic organisation of frontal appendages of different radiodont families. **a** Amplectobeluidae and Anomalocarididae. One marks first podomere in distal articulated region, bearing hypertrophied endite. Five marks podomere where endite is longer than on podomere 3 in Amplectobeluidae, but not Anomalocarididae. **b** Tamisiocarididae. One marks first podomere in distal articulated region. **c** Hurdiidae. One marks first podomere in distal articulated region, 5 marks most distal podomere bearing elongate endite. **d** Attachment of paired endites to podomere in Amplectobeluidae, Anomalocarididae, and Tamisiocarididae (cross-sectional view). **e** Single blade-like endite attached to podomere in Hurdiidae (cross-sectional view)

Both the shaft and distal articulated region can show articulations between podomeres, however those in the shaft are generally more weakly defined [24], and in some cases the shaft is not preserved at all in any known specimen of a species (e.g. *Amplectobelua stephenensis* [22]). In amplectobeluids, and in some cases in anomalocaridids, there is an angle on the dorsal surface between the shaft and distal articulated region (θ in Fig. 1a; [24]), and in both amplectobeluids and anomalocaridids the endite on the first podomere in the distal articulated region is often enlarged or hypertrophied, meaning that the shaft can be identified as all podomeres proximal to this endite.

**Fig. 2:**
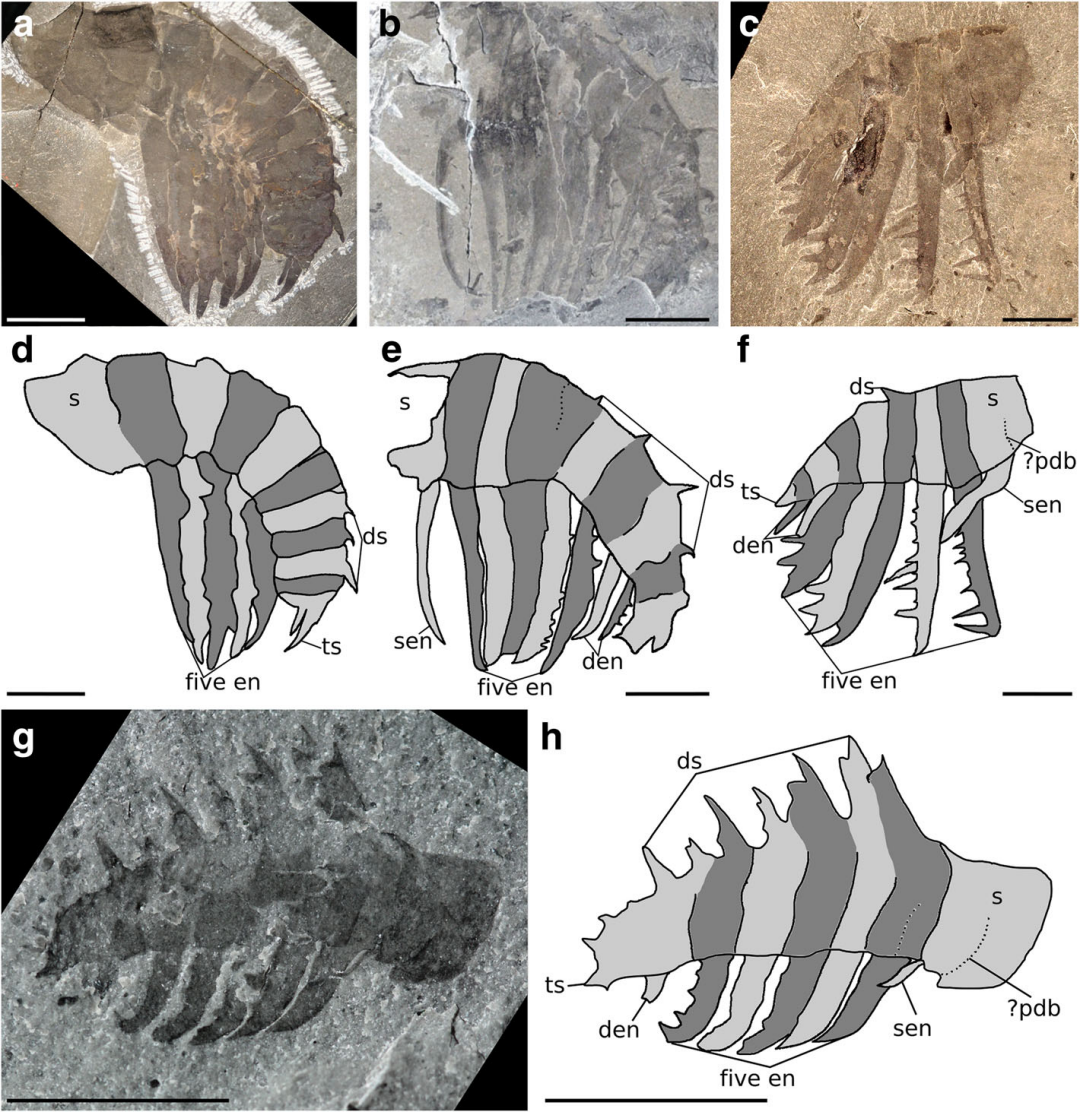
Hurdiid frontal appendages bearing auxiliary spines. **a** and **d** *Peytoia nathorsti* from the Burgess Shale, British Columbia, Canada, USNM 240984. **b** and **e** ?*Peytoia* from the Burgess Shale, British Columbia, Canada, ROM 59508 (*?Laggania* of [22]). **c** and **f**. *Hurdia* from the Burgess Shale, British Columbia, Canada, ROM 60048. **g** and **h** *Stanleycaris hirpex* from Stephen Formation, British Columbia, Canada, ROM 59975. Abbreviations: den, distal endites; ds, dorsal spines; five en, five subequal endites on first five podomeres of distal articulated region; pdb, podomere boundary; s, shaft; sen, shaft endite. Scale bars = 10 mm

**Fig. 3:**
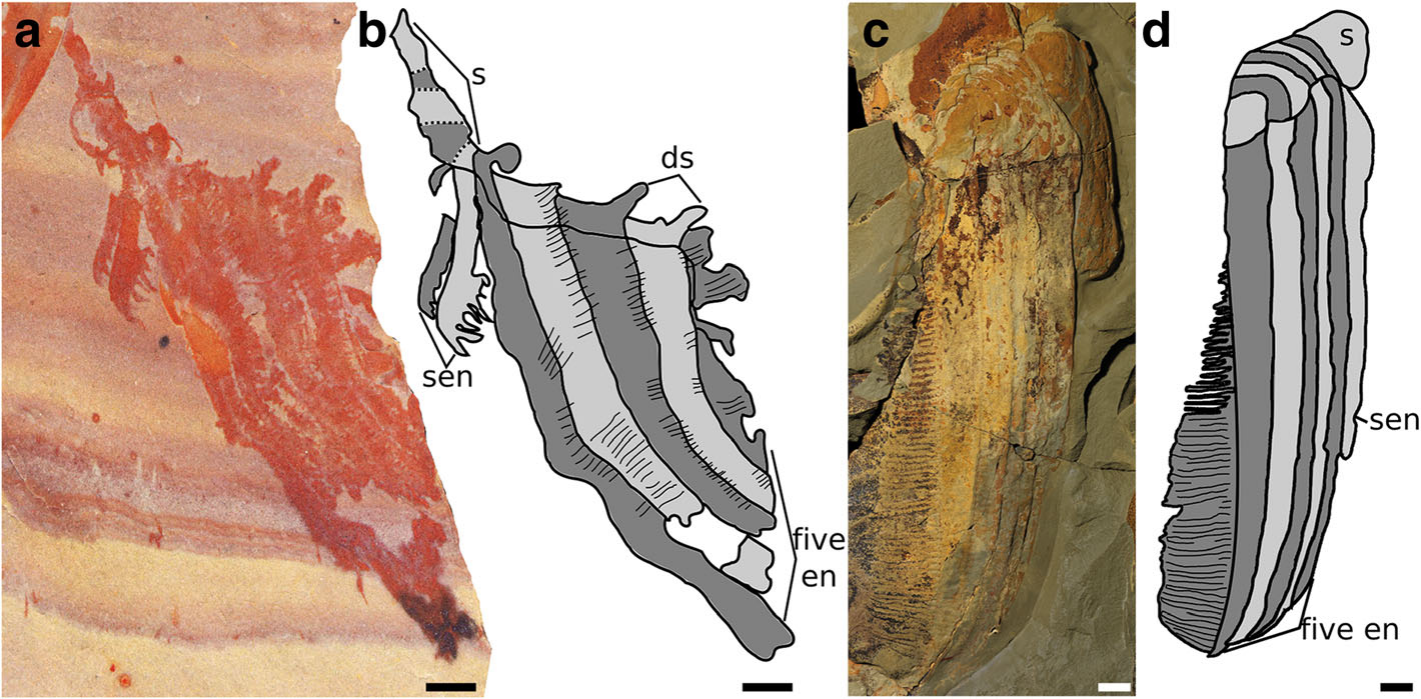
Hurdiid frontal appendages bearing setae (only most prominent setae drawn). **a** and **b** *Pahvantia hastata* from the Wheeler Formation, Utah, USA, KUMIP 314089. **c** and **d** *Aegirocassis benmoulae* from the Fezouata Formation, Morocco, YPM 527123. Abbreviations: ds, dorsal spines; five en, five subequal endites on first five podomeres of distal articulated region; s, shaft; sen, shaft endite. Scale bars: **a**, **b** = 2 mm; **c**, **d** = 10 mm

There is often, but not always, an endite present on the shaft, and in rare cases more than one endite is known, as seen in *Pahvantia hastata, Paranomalocaris multisegmentalis,* and *Ramskoeldia platyacantha* [16, 24, 26]*.* The distal shaft endite contrasts to the endites in the distal articulated region in its location and morphology. The distal shaft endite generally protrudes from the distalmost point of the podomere’s ventral surface, whereas the endites in the distal articulated region arise from the midpoint of the podomere’s ventral surface, halfway between the two podomere boundaries. Shaft endites are also usually distinct in morphology from the endites in the distal articulated region. The shaft endite is reduced to a simple or straight spine in *Anomalocaris canadensis, An. kunmingensis, An. saron, Hurdia, Lyrarapax trilobus, Peytoia?, Ramskoeldia consimilis, Stanleycaris hirpex,* and *Tamisiocaris borealis* (Fig. 2b, c, e–h) [13, 23, 24, 27]. In *An. briggsi*, *Laminacaris*? sp. (Kinzers Formation), and *Pahvantia hastata*, it is recurved and bears auxiliary spines (Fig. 3a, b) [16, 28, 29], and in *Aegirocassis benmoulai,* it likely bore posterior-facing auxiliary spines [10]. The shaft endite in hurdiids commonly points more distally to those in the distal articulated region. This can be seen in *Hurdia* and *Stanleycaris* (sen, Fig. 2c, f-h). Similarly in *Peytoia?* the shaft endite is recurved distally, so at the point closest to the podomere it is orientated more proximally than other endites, and at the point furthest from the podomere it is orientated more distally (sen, Fig. 2b, e).

As no known hurdiid bears a hypertrophied endite on the first podomere in the distal articulated region, and as an angle on the dorsal surface between shaft and distal articulated region is rarely present in this family, the most reliable way to determine the shaft endite for hurdiids is to examine the location (distal margin of a podomere), morphology (distinct from the endites on more distal podomeres), and/or orientation (more distally pointing than the endites in the distal articulated region) of endites.

## Hurdiid and tamisiocaridid frontal appendages

Each of the four radiodont families has a recognisable and distinct frontal appendage morphology and arrangement of endites (Table 1 in [28]; Fig. 1). Hurdiidae, a family including the genera *Aegirocassis*, *Hurdia, Pahvantia, Peytoia* and *Stanleycaris*, as well as questionably the early Devonian animal *Schinderhannes* [2, 10, 15, 16, 18, 23, 30] (Figs. 2 and 3) possess frontal appendages very different even to those of members of its sister-family, Tamisiocarididae, which currently includes two taxa: *Anomalocaris briggsi* and *Tamisiocaris borealis* [10–12, 16, 17, 23, 29].

**Table 1:** Comparison of frontal appendage characters in select hurdiid and tamisiocaridid genera

	Shaft endite(s)	Endite morphology and orientation (Fig. 1d, e or Fig. 6b)	Auxiliary spines/ setae	#pd with elongate endites in d.a.r.	Shape of arthrodial membrane	Refs
Hurdiidae						
*Aegirocassis*	Elongate, bears proximally facing auxiliary spines	1e	Dist	5	Straight	[10]
*Hurdia*	Reduced, distally pointing	1e	Dist	5	Straight	[18, 24, 29]
*Pahvantia*	Two, recurved, with auxiliary spines on distal margin	1e	Dist	5	Straight	[16]
*Peytoia*	Absent	1e	Dist	5	Straight	[29]
*Peytoia*? (?*Laggania* of Daley & Budd 2010)	Elongate, with no auxiliary spines	1e	Dist	5	Straight	[22]
*Stanleycaris*	Very reduced, distally pointing	1e	Dist	5	Straight	[30, 37]
*Ursulinacaris*	Elongate, with no auxiliary spines, distally pointing	6b	Dist	5	Straight	this study
Tamisiocarididae						
*Tamisiocaris*	Elongate spine with no auxiliary spines	1d	Dist and prox^a^	17	Triangular	[11, 23]
*Anomalocaris briggsi*	Wide and short, bearing auxiliary spines on distal and proximal margins	1d	Dist and prox	12	Triangular	[7]

^a^No auxiliary spines are known in *Tamisiocaris* from the Kinzers Formation, however this could be the result of taphonomic removal [28]. *Abbreviations*: *d.a.r* distal articulated region, *dist* distal margin of endite, *prox* proximal margin of endite, *Refs* references

The frontal appendages of all previously described hurdiid taxa bear unpaired blade-like endites of subequal length on the first five podomeres in the distal articulated region (Fig. 1c, e; five en, Figs. 2 and 3). Each of these endites is longer than the height of the podomere to which it attaches, and bears auxiliary spines or setae on its distal margin only. Podomeres distal to the five subequal endites are reduced in height and in most cases width (although this is not always clear). In addition, some taxa—*Hurdia, Stanleycaris,* and *Peytoia?* from the Burgess Shale (*?Laggania* of [22], a distinct taxon from *Peytoia nathorsti*)—bear additional shorter endites in this region (den, Figs. 2 and 3). Shaft endites are known in both filter-feeding taxa, *Aegirocassis* and *Pahvantia* (sen, Fig. 3) [10, 16], as well as *Hurdia, Stanleycaris,* and *Peytoia*? (sen, Fig. 2).

Just as in hurdiids, tamisiocaridid endites are longer than the podomere to which they attach, and are subequal in length (Fig. 1b, c). However, unlike hurdiids, tamisiocaridids bear endites on every podomere in the distal articulated region, except the terminal podomere (Fig. 1b). In addition, tamisiocaridid endites are paired and slender, and bear auxiliary spines on both distal and proximal margins (Fig. 1b, d) [7, 23].

Here we describe a new genus of hurdiid, *Ursulinacaris*, known only from frontal appendages, possessing elongate endites with auxiliary spines on the distal margin only on the first five podomeres in the distal articulated region. However, unlike all other hurdiids these endites are slender and paired, as in tamisiocaridids. These new data allow the presence of a single blade-like endite per podomere to be identified as a hurdiid autapomorphy. Thus, *Ursulinacaris grallae* provides crucial information on the sequence of character acquisition for hurdiid frontal appendages, a body part of great importance for evolutionary and ecological studies of this family, and radiodonts as a whole.

## Non-frontal appendage features in hurdiids

In some hurdiid taxa additional features beyond the frontal appendages are known. Mouthparts have been described for *Hurdia, Peytoia* and *Stanleycaris*. These consist of four large plates arranged around a square or rectangular opening, separated from one another by seven smaller plates. Triangular spines line the central opening, and in *Hurdia* additional rows of spines are present within [30, 31].

A tripartite frontal carapace of a central element (homologous to the anterior sclerite in other groups [32]) and paired lateral elements is known in *Aegirocassis, Hurdia,* and *Pahvantia* [10, 16, 18].

Where the remainder of the body has been described, for *Aegirocassis, Hurdia* and *Peytoia*, two sets of flaps (dorsal and ventral) are present, distinguishing hurdiids from all other radiodonts (which have only one set of flaps) [10].

## Materials and methods

Isolated radiodont frontal appendages have been known for over 20 years from the Mount Cap Formation, alongside bivalved euarthropods and segmented coprolites [33, 34]. Four appendages, preserved as flattened carbon films, were studied from this deposit. Unusually for BST assemblages, the Mount Cap Formation was formed in an intracratonic basin cut off from the ocean by the Mackenzie Arch [34]. Trilobite biostratigraphy identifies a *Glossopleura walcotti* zone age (Miaolingian, Wuliuan) for this biota [34].

The single radiodont frontal appendage, preserved as a flattened carbon film, reported herein is the first soft-bodied fossil known from the Jangle Limestone Member of the Carrara Formation. The Jangle Limestone Member preserves a marine environment with the fauna dominated by trilobites [35]. Trilobite biostratigraphy identifies the level from which the frontal appendage was obtained as the *Mexicella mexicana* zone (Miaolingian, Wuliuan), slightly older than the Mount Cap Formation specimens, which correlate with a level at the very top of the Jangle Limestone Member [35].

Specimens were photographed using a Canon EOS 500D digital SLR camera with Canon EF-S 60 mm Macro Lens, controlled for remote shooting using EOS Utility 2.

### Terminology and orientation

The term ‘shaft’ is used (following [24]) to identify the proximal podomeres of the appendage attaching them adjacent to the mouthparts. It is equivalent to ‘peduncle’ used in other studies (e.g. [16, 25]). The term ‘endite’ is used (following [36]), and is equivalent to ‘ventral spine’ and ‘ventral blade’ used in other studies (e.g. [37, 38]). Measurements of the length of the whole appendage and width of individual podomeres refer to the dimension along the proximal-distal axis. Measurements of the height of individual podomeres and length of endites refer to the dimension along the dorso-ventral axis.

## Results

### Systematic Palaeontology

Panarthropoda Nielsen, 1995 [39]

Radiodonta Collins, 1996 [40]

Hurdiidae Lerosey-Aubril and Pates, 2018 [16]

#### Genera

*Aegirocassis* Van Roy, Daley, & Briggs 2015, *Hurdia* [10] Walcott 1912 [41]*, Pahvantia* Robison & Richards 1981 [42]*, Peytoia* Walcott 1911 [43]*, Stanleycaris* Pates, Daley, & Ortega-Hernández 2018 [44], and *Ursulinacaris* nov. Questionably *Schinderhannes* Kühl, Briggs, & Rust 2009 [15].

#### Remarks

A number of phylogenetic analyses [10, 12, 16, 17, 23] have recovered *Schinderhannes* within a monophyletic Hurdiidae, so it is questionably included here pending a redescription revealing more details of, for example, the mouthparts. The frontal appendage of *Schinderhannes* bears a number of similarities with hurdiids; specifically, an elongate shaft endite and five subequal endites in the distal articulated region. The morphology of the mouthparts is not well known (only that they are radially arranged) and flaps are present along the trunk. *Schinderhannes* also possesses a number of characters not known in other radiodonts, such as putative tergites and biramous trunk appendages. As a result, the first phylogenetic analysis to include this taxon placed it between radiodonts and deuteropods (sensu [32]) [15].

As well as the named taxa (above), *Peytoia*? from the Burgess Shale (?*Laggania* of [22]) is here treated as a member of Hurdiidae, distinct from *Peytoia*. This is based on the presence of two features in *Peytoia*? not known in *Peytoia*: an elongate and recurved shaft endite, and additional shorter endites in the distal articulated region (den, sen, Fig. 2b, e). *Ursulinacaris* gen. Nov.

#### Etymology

From Latin: ‘ursulina’, the diminutive adjective of ‘ursa’, meaning ‘from little bear’, a reference to the locality of the holotype; and ‘caris’ meaning ‘crab’, a commonly used suffix for marine euarthropods. Gender feminine.

#### Type species

*Ursulinacaris grallae* sp. nov.

#### Diagnosis

Radiodont with frontal appendage consisting of at least 12 podomeres, including two in the shaft, and at least 10 in the distal articulated region; distalmost shaft podomere bears a single elongate endite orientated distally; elongate and slender endites in the distal articulated region are paired and with small auxiliary spines along their distal margin; distal podomeres flexed ventrally, are reduced in height, but increased in width; podomeres 8 and 9 bear simple endites without auxiliary spines; small dorsal spines present at the distal margin of all podomeres in the distal articulated region.

*Ursulinacaris grallae* sp. nov.

Figures 4, 5 and 6

**Fig. 4:**
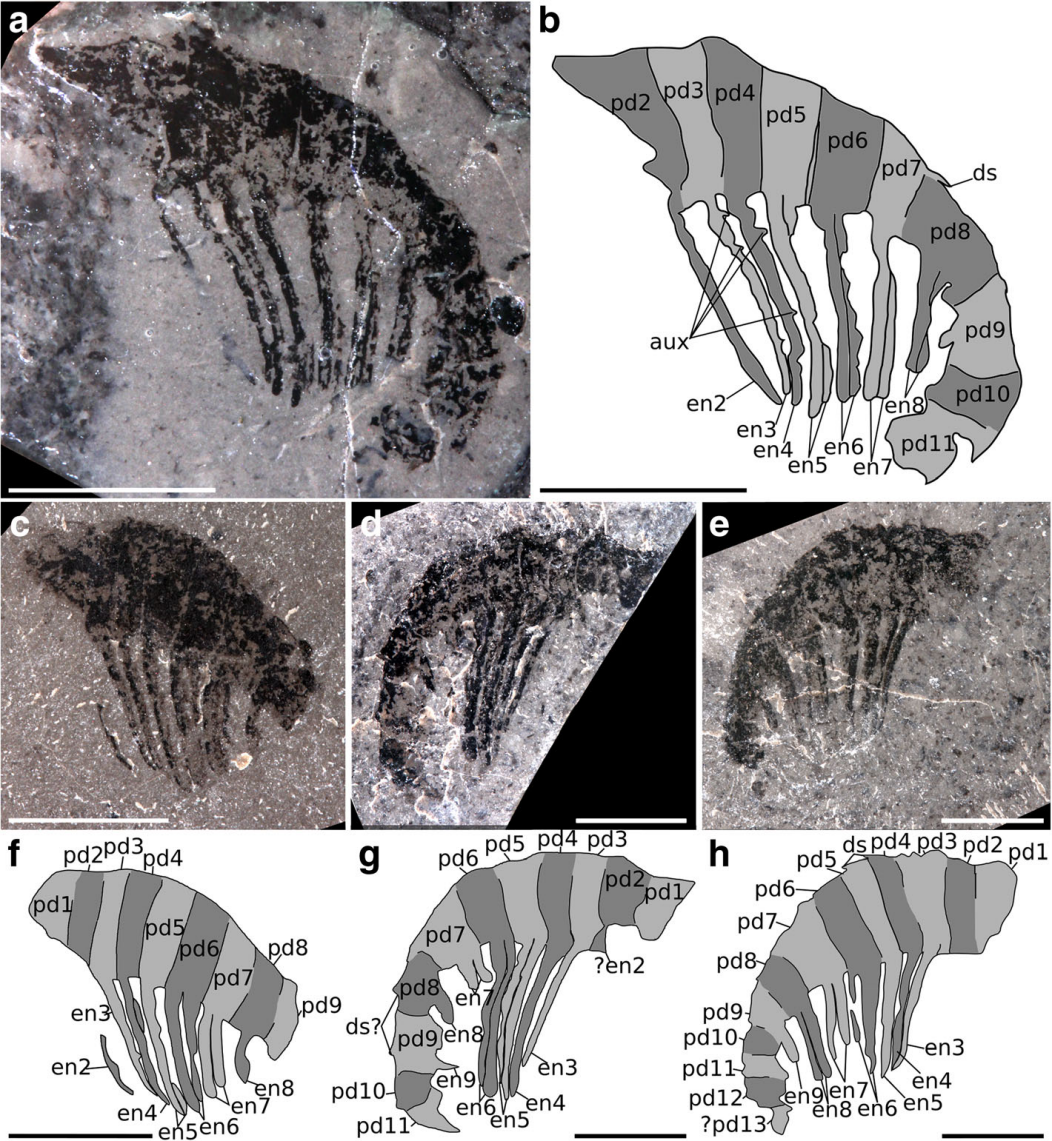
*Ursulinacaris grallae* appendages from the Mount Cap Formation, Northwest Territories, Canada. **a** and **b** GSC 140185. **c** and **f** GSC 135494a. **d** and **g** GSC 140186a. **e** and **h** GSC 140184. Abbreviations: aux, auxiliary spine; ds, dorsal spine; en, endite; pd, podomere. Scale bars = 5 mm

**Fig. 5:**
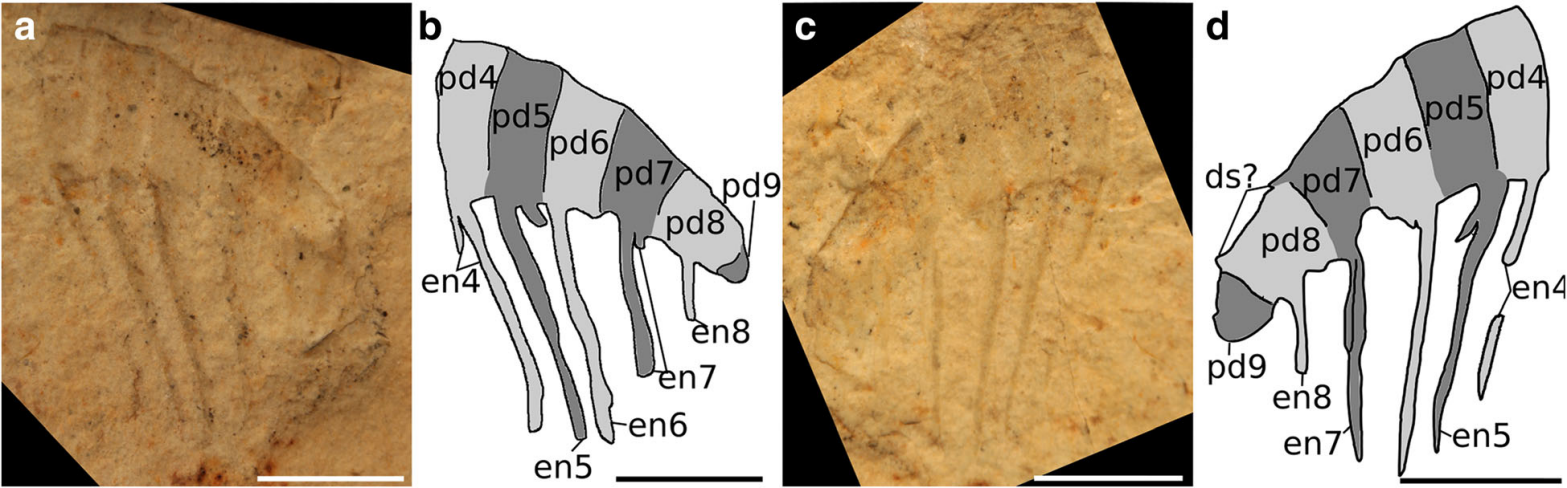
*Ursulinacaris grallae?* partial appendage from the Jangle Limestone Member, Carrara Formation, Nevada, USA. KUMIP 492945. **a** and **b** part. **c** and **d** counterpart. Abbreviations: en, endite; pd., podomere. Scale bars = 5 mm

**Fig. 6:**
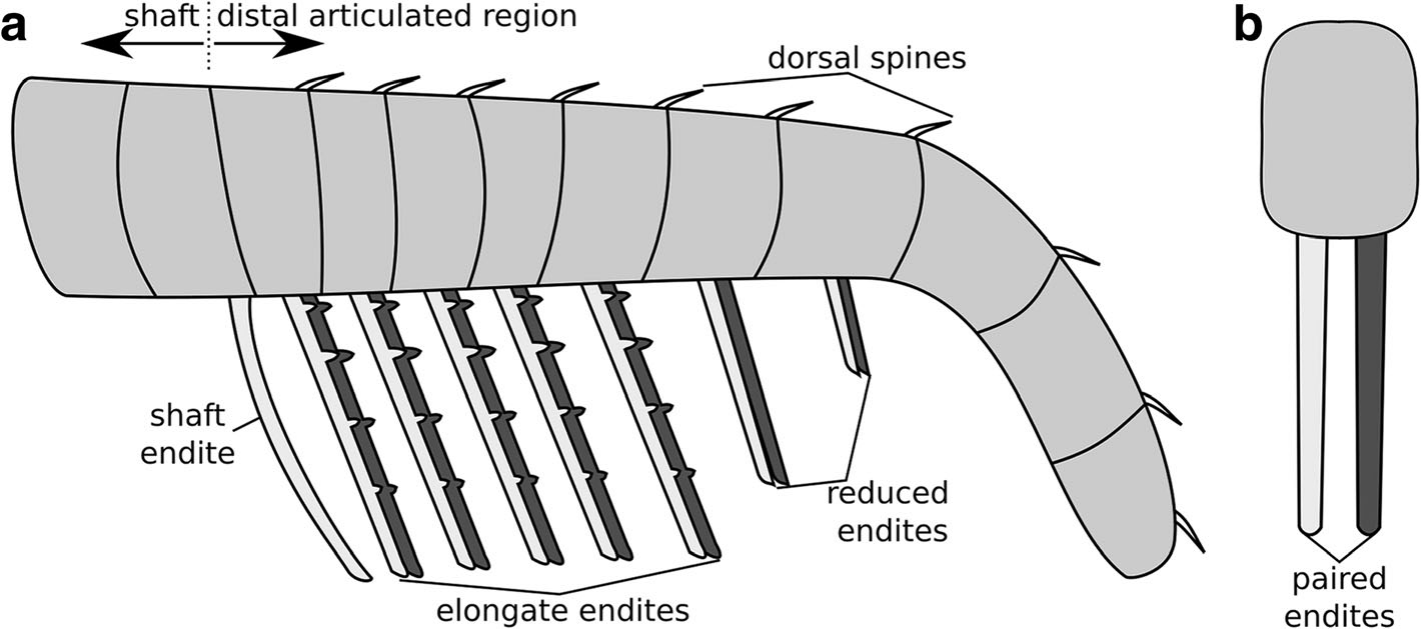
Reconstruction of *Ursulinacaris grallae* frontal appendage, with distal podomeres unflexed. **a** lateral view. **b** cross-sectional view

v. 1996 ‘anomalocarid claw’; Butterfield & Nicholas, p. 895, fig. 2.2

v. 2011 ‘Anomalocaridid claw’; Harvey & Butterfield, p. 168, fig. 3a

#### Etymology

From Latin: ‘*grallae’* meaning ‘stilts’, referring to the long and slender ventral endite morphology, and pairing of endites.

#### Referred specimens

GSC 140185 (counterpart GSC 140185a) (holotype); GSC 135494 (counterpart GSC 135494a); GSC 140184, GSC 140186 (counterpart GSC 140186a) and questionably a partial frontal appendage KUMIP 492945 (part and counterpart).

#### Locality and horizon

In 64° 28.77′ north, 126° 47.291′ west, *Glossopleura walcotti* zone, Little Bear biota, Mount Cap Formation, Northwest Territories, Canada (GSC specimens); questionably from 36° 23′ north, 120° 0′ west, *Mexicella mexicana* zone, Jangle Limestone Member, Carrara Formation, North face of Mount Montgomery, Last Chance Range, Nye County, Nevada, USA (KUMIP 492945).

#### Diagnosis

As for genus

#### Description

This taxon is known from four complete or nearly complete frontal appendages (length of dorsal surface between 13 and 24 mm) from the Mount Cap Formation (Little Bear biota; Fig. 4), and one questionable partial specimen (length of dorsal surface 14 mm) from the Carrara Formation (Jangle Limestone Member; Fig. 5). The shaft is composed of two podomeres, approximately twice as tall as wide (pd1, pd2, Fig. 4c–h), and an elongate curved endite extends from the distalmost portion of the shaft, angled towards the distal end of the appendage (en2, Fig. 4). There is no evidence that this endite is paired. Immediately distal to the shaft are five podomeres (pd3–7) at least twice as high as wide. Each of these bears a pair of thin, straight endites, which attach to the podomere at the midpoint of the ventral surface. Endites are approximately twice as long as the height of the podomere to which they attach (en3–7, Fig. 4). Only four of these podomeres are preserved in the Jangle Limestone specimen, with one long endite visible per podomere alongside the base of its pair (en4–7, Fig. 5). These endites attach separately to the podomeres and run parallel to the sagittal plane of the appendage, best seen in the obliquely preserved GSC 135494a (en6, en7, Fig. 4c, f). Small auxiliary spines are present on the distal margin of the endites, most visible on endites 3 and 4 in GSC 140185 (aux, Fig. 4a, b), although none are visible on the partial Carrara Formation specimen (Fig. 5), or the other three Mount Cap Formation specimens (Fig. 4c–h). Podomere 8, and more distal podomeres (when preserved), are shorter and appear wider than more proximal podomeres. The endites on podomere 8 are also paired, but are slightly shorter than the endites on podomeres 3–7 (en8, Figs. 4a, b, e, h and 5), it is not clear whether the endite on podomere 9 is paired (en9, Fig. 4d, e, g, h). The total number of podomeres cannot be counted with certainty; however, at least five (possibly six) podomeres are present (pd8–12,? pd13, Fig. 4e, h) distal to the five podomeres bearing elongate endites (pd3–7). Small, thin, dorsal spines are rarely preserved, but can be seen at the distal margin of some podomeres (ds and ds?, Fig. 4a, b, d, e, g, h). The presence of terminal spines cannot be confirmed owing to the poor preservation of the distal region in all specimens.

#### Remarks

*Ursulinacaris* frontal appendages have an overall organisation very similar to all other hurdiids, with the proximal five podomeres in the distal articulated region bearing elongate endites approximately twice as long as the podomeres height, and auxiliary spines only on the distal margin (Figs. 1c, 2 and 3). Unlike for amplectobeluids, anomalocaridids, and those hurdiids with a two-podomere shaft (Fig. 2), the articulation between podomeres in the shaft (pd1 and pd2) in *Ursulinacaris* is nearly as well defined as those in the distal articulated region (pd3 and more distal podomeres). The shaft is identified by the presence of an endite (en2) at or close to its distal margin with a subtly different morphology from that of the endites in the distal articulated region. There is no evidence that the shaft endite is paired or bears auxiliary spines. This is unlike the elongate endites in the distal articulated region where at least the base of a pair is visible in most cases (although it should be noted that the proximal podomeres in the distal articulated region for the specimen where the shaft is clearest (Fig. 4a, b) do not clearly show paired endites) and auxiliary spines are visible on some endites (though only in one specimen, Fig. 4a, b). In addition, the endite on podomere 2 (the endite-bearing shaft podomere) has a different angle of attachment to the podomere, pointing more distally, and has slightly more distal curvature (compare en2 to other endites in Fig. 4b and Fig. 4f). Unlike in all other hurdiids where shaft endites have been recognised, the shaft endite in *Ursulinacaris* does not appear to be shorter than the endites of the distal articulated region. The podomeres in the distalmost region are reduced in height, and flexed ventrally, a feature often seen in the appendages of *Peytoia* and *Peytoia*? (Fig. 2a, b, d, e). The endites on pd8 and pd9 are shorter than those on pd3–7, just as the distalmost endites in *Hurdia, Stanleycaris* and *Peytoia?* are shorter than the five elongate subequal endites (Fig. 2b, c, e–h), but unlike in these taxa, the distal podomeres in *Ursulinacaris* appear to be wider than the more proximal ones. The number of endites per podomere and morphology of endites differs between *Ursulinacaris* (paired, thin, straight) and other hurdiids (unpaired, thick, recurved blade-like). The long endites bear a resemblance to tamisiocaridid endites (*Anomalocaris briggsi* and *Tamisiocaris borealis* [7, 23]), as they are thin and paired. They differ however as they are not present on every podomere, bear auxiliary spines on their distal margins only, and do not diverge from the sagittal plane of the appendage distally as much as *Tamisiocaris* endites (compare Fig. 1d to Fig. 6b). As summarized in Table 1, the characteristics of the frontal appendage of *Ursulinacaris grallae* warrant its assignment to the family Hurdiidae. Within Hurdiidae *Ursulinacaris* is most similar to *Peytoia?* from the Burgess Shale (Fig. 2b, e), as both bear an elongate shaft endite, and reduced endites in the distal region of the appendage.

## Discussion

### Paired endites in all hurdiids?

Given the discovery of paired endites in the hurdiid *Ursulinacaris*, it is worth assessing whether any previously described hurdiid frontal appendages might also possess paired endites, as the taphonomic removal of fine structures, particularly spines and endites, is known to occur in compressed carbonaceous impression fossils [27]. In *Anomalocaris canadensis* from the Burgess Shale, many specimens only have one of the paired endites visible per podomere, with the second one being obscured by other anatomical structures, completely removed, or preserved in a different plane to the rest of the fossil ([27]: figs. 1, 8.1, 10.1, 10.2, 10.5). Even along the length of one individual *Anomalocaris* appendage, there can be variation in whether two, one or zero endites are visible on each podomere ([27]: figs. 11.1, 11.4, 11.5]. This can be linked to the orientation of specimens relative to the bedding plane during compression, with slightly oblique preservation often resulting in the preservation of both paired endites ([27]: fig. 12.3–12.7). Hurdiids can also be preserved at oblique angles to bedding, with documented taphonomic effects including changes to the apparent thickness of the blade-like endite and the degree of curvature, but not the taphonomic removal of an entire endite ([38]: fig. 8). The examination of 725 *Hurdia* and *Peytoia* frontal appendages [29], 41 *Peytoia*? frontal appendages [19], and 37 *Stanleycaris* frontal appendages [30] from the Burgess Shale revealed no indication of paired ventral spines in any specimen, despite these appendages showing a variety of preservation states and orientations, including with endites widely splayed out with clear space in between each endite ([29]: fig. 12C–E). There is also no indication of paired endites in specimens of these three hurdiid taxa from other localities, including *Peytoia infercambriensis* from Poland [8], *Peytoia* sp. cf. *P. nathorsti* from the Balang Formation in China [45], *Peytoia nathorsti* from the Wheeler Formation [38], *Hurdia* from the Spence Shale [29, 38], and *Stanleycaris* from the Wheeler Formation in Utah [37, 44]. This suggests that paired endites were not present in *Hurdia*, *Peytoia*, or *Stanleycaris*. In contrast, every one of the five known specimens of *Ursulinacaris* have both endites in the pair visible in for at least one podomere. Frontal appendages of the other two previously described hurdiid genera, *Aegirocassis* from the Fezouata Biota [10] and *Pahvantia* from the Wheeler Formation [16], are known in much smaller numbers, however these specimens also show no indication of paired endites. It should be noted that *Pahvantia* does show two rows of setae per endite [16]. This may be indicative of the plesiomorphic paired endite condition as seen in *Ursulinacaris*, where each endite has one row of auxiliary spines, resulting in two rows of auxiliary spines per podomere. All other hurdiids bear a single row of auxiliary spines on their blade-like endites [8, 18, 22, 29, 30, 37], or in the case of *Aegirocassis*, a single row of setae [10].

### A hurdiid with paired endites

*Ursulinacaris grallae* provides information on the sequence of acquisition of characters in the hurdiid frontal appendage. As a hurdiid with paired endites, this new taxon demonstrates that the presence of a single, blade-like endite on podomeres in the distal articulated region (Fig. 1e) is the autapomorphic condition for the family. Two other frontal appendage characters known in all hurdiids, the presence of elongate endites on the first five podomeres in the distal articulated region only (five en, Figs. 2 and 3), and auxiliary spines or setae along the distal margin of endites only, predate the blade-like endite.

### Problems with resolving internal hurdiid relationships with phylogenetic methods

Hurdiid appendages have a consistent arrangement (Fig. 1c), but vary in the relative length and thicknesses of endites, morphology and robustness of dorsal spines, the morphology of the shaft endite, number of shaft endites, and morphology of auxiliary spines (Figs. 2, 3 and 6). Two taxa, *Aegirocassis* and *Pahvantia*, possess setae on the distal margin of endites, in place of auxiliary spines present in all other taxa (Fig. 3) [10, 16]. Crucially all hurdiids show unique characteristics in their frontal appendage morphology when compared to other members of the family, with no apparent sequential acquisition of characters that would provide resolution from a phylogenetic analysis. Hurdiids differ also in the anatomy of other body parts, such as the number of body metameres, and morphology of frontal carapaces and lateral flaps, but these features are known in under half of hurdiid taxa (Table 2). Because the frontal appendages are the most character-rich body part used in phylogenetic analyses, and because there are very few shared character states between the few genera with known body, flaps and carapaces, the hurdiid clade of radiodont phylogenetic analyses tends to be poorly resolved (e.g. [16, 17]). The identification of paired endites in the frontal appendage of *Ursulinacaris*, a hurdiid, will therefore inform future radiodont phylogenetic analyses. This description, alongside additional new hurdiid taxa, will help to resolve both internal hurdiid relationships and the relationship between hurdiids and other radiodont families, by providing novel frontal appendage character combinations which may allow hurdiid synapomorphies and autapomorphies to be distinguished.

**Table 2:** Body parts known in hurdiids. Taxa analysed in the most recent phylogenetic analysis of radiodonts [16] in bold

	Frontal appendages	Mouthparts	Body and flaps	Carapace
***Aegirocassis***	**X**		**X**	**X**
***Fezouata hurdiid***	**X**			
*Hurdia triangulata*	X	X	X	X
***Hurdia victoria***	**X**	**X**	**X**	**X**
***Hurdia*** **sp. B (Burgess)**	**X**			
***Hurdia*** **sp. B (Spence)**	**X**			
***Hurdia cf. victoria*** **(Spence)**	**X**			
***Pahvantia***	**X**			**X**
*Peytoia infercambriensis*	X			
***Peytoia nathorsti***	**X**	**X**	**X**	**X**
***Peytoia*** **sp. (Balang)**	**X**			
*Peytoia?* (Burgess)	X			
***Schinderhannes***	**X**	**X**	**X**	
***Stanleycaris***	**X**	**X**		
*Ursulinacaris*	X			

## Conclusion

The description of paired endites in a new genus of hurdiid, *Ursulinacaris*, shows that this character is present in all families of radiodont. All other known hurdiids are still thought to have had unpaired blade-like endites. The character combination present in *Ursulinacaris* suggests that the blade-like unpaired endite known in all other hurdiids represents an innovation obtained after hurdiid frontal appendages had developed the distinctive five elongate endite-bearing podomeres in the distal articulated region, and the presence of auxiliary spines on the distal margin of endites only. This new radiodont, *Ursulinacaris*, along with additional future descriptions of new hurdiid taxa, will provide crucial data for resolving the sequence of character acquisitions in the radiodont frontal appendage, as well as inter- and intrafamily relationships in future phylogenetic analyses of the group.

## Data Availability

Fossil material used in this study is accessioned at the GSC and KUMIP. This published work and the nomenclatural acts it contains have been registered in ZooBank. Manuscript LSID: urn:lsid:zoobank.org:pub:AEDA6CD0-4839-4667-B481-C64B5218C471. Genus *Ursulinacaris*: urn:lsid:zoobank.org:act:1E7FA414-B176-478D-9A99-97F977525DE4. Species *Ursulinacaris grallae*: urn:lsid:zoobank.org:act:922BBC29-18E3-4CF1-ABBA-4A361F3FF669

## Abbreviations

d.a.r: distal articulated region
en: endite
GSC: Geological Survey of Canada, Ottawa, Ontario, Canada
KUMIP: Division of Invertebrate Paleontology, Biodiversity Institute, University of Kansas, Lawrence, Kansas, USA
pd: podomere
ROM: Royal Ontario Museum, Toronto, Ontario, Canada
USNM: National Museum of Natural History, Washington DC, USA
YPM: Yale Peabody Museum, New Haven, Connecticut, USA
